# Early intervention for children with developmental disabilities in low and middle-income countries – the case for action

**DOI:** 10.1093/inthealth/ihaa044

**Published:** 2020-08-11

**Authors:** Tracey Smythe, Maria Zuurmond, Cally J Tann, Melissa Gladstone, Hannah Kuper

**Affiliations:** International Centre for Evidence in Disability, London School of Hygiene & Tropical Medicine, London, WC1E 7HT, UK; International Centre for Evidence in Disability, London School of Hygiene & Tropical Medicine, London, WC1E 7HT, UK; Maternal, Adolescent, Reproductive and Child Health Centre, London School of Hygiene & Tropical Medicine, London, WC1E 7HT, UK; MRC/UVRI & LSHTM Uganda Research Unit, Entebbe, Uganda; Institute of Translational Medicine, University of Liverpool, Liverpool, L69 7TX, UK; International Centre for Evidence in Disability, London School of Hygiene & Tropical Medicine, London, WC1E 7HT, UK

**Keywords:** child development, children with disabilities, developmental disabilities, disability studies, early intervention

## Abstract

In the last two decades, the global community has made significant progress in saving the lives of children <5 y of age. However, these advances are failing to help all children to thrive, especially children with disabilities. Most early child development research has focussed on the impact of biological and psychosocial factors on the developing brain and the effect of early intervention on child development. Yet studies typically exclude children with disabilities, so relatively little is known about which interventions are effective for this high-risk group. In this article we provide an overview of child development and developmental disabilities. We describe family-centred care interventions that aim to provide optimal stimulation for development in a safe, stable and nurturing environment. We make the case for improving opportunities for children with developmental disabilities to achieve their full potential and thrive, including through inclusive early childhood development intervention. Finally, we call for the global research community to adopt a systematic approach for better evidence for and implementation of early interventions for children with developmental disabilities in low-resource settings.

## Introduction

Substantial global progress has been made in reducing child deaths since 1990 and the mortality rate of children <5 y of age has decreased in all world regions. However, non-communicable morbidities and disabilities have not been addressed to the same extent. This review discusses the urgency of taking actions to narrow the inequality gap in early childhood developmental care, especially for the 53 million children <5 y of age living with disabilities and developmental disorders such as epilepsy, intellectual disability, sensory impairments, autism spectrum disorder and attention deficit hyperactivity disorder.^1^ A focus on supporting children with disabilities to thrive during their early years is important, as this period is critical for maximising their development. Furthermore, under the United Nations Convention of Rights for a Child and the United Nations Convention of the Rights of Persons with Disabilities, governments are duty-bound to provide early years services that are inclusive of and available to all children.^2,3^ This article will describe child development and developmental disabilities and make the case for which equitable early childhood development (ECD) interventions may be optimal for helping children with developmental disabilities to achieve their potential.

## Child development

Early childhood is a period of great opportunity for optimum brain growth, but it is also a period of vulnerability. Development in language, cognition, motor and socio-emotional domains occurs rapidly in these first years. These areas of development do not operate or develop in isolation, but enable each other and mutually interact as the child learns to become more independent. For instance, as a child learns to see, she will increasingly reach for and play with objects and thereby develop motor skills and coordination. Biological, psychosocial^4,5^ and environmental factors also crucially affect the structure and functioning of the brain as it is developing.^6^ For example, if a child experiences adequate nutrition and is provided with opportunities to play, she may progressively explore her environment and interact with her caregiver and by doing so, reinforce her psychosocial development. Furthermore, the time period when these factors influence brain growth are critically important, as there are particular early windows of opportunity that if not harnessed, may prevent optimal brain development and lifelong well-being.^7^

It is increasingly apparent that optimal early child development has lifetime beneficial consequences for educational achievement, adult productivity and population health.^8–10^ Conversely, exposure to biological and psychosocial risks negatively affects the developing brain and compromises the development of children.^5^ Many structural factors determine these early child circumstances. These factors include a lack of nurturing care (nutrition, stimulation, good health) in the early years, as well as inadequate cognitive and psychosocial stimulation.^5,11^ Children <5 y of age in low- and middle-income countries (LMICs) may be particularly at risk of poor development due to poor health and nutrition.^7^

Child development can be encouraged through intervention in early childhood.^11^ A number of mutually important elements are needed for maximising children's development. These include supporting responsive relationships, reducing sources of stress in the lives of children and families, building executive function and self-regulation skills and reinforcing contexts in which learning is most achievable across all developmental domains.^12,13^ ECD interventions work to improve development through integrating family support, health, nutrition and educational services and providing direct learning experiences to young children and families.^14^

The strategic focus of the World health Organization (WHO), United Nations Children's Fund (UNICEF) and World Bank ‘Nurturing Care Framework’ is therefore timely.^15–17^ This action plan provides a framework for helping children survive and thrive through five strategic actions—lead and invest, focus on families and their communities, strengthen services, monitor progress and use data and innovate—and thereby aims to transform health and human potential. We know that urgent action is necessary to improve early childhood outcomes and ensure that all children reach their full potential as adults. Children with developmental disabilities must be included in this agenda, as they are a marginalised group with additional and specific needs and will otherwise be left behind.

## Developmental disability

Developmental delay and developmental disability are two distinct concepts. Developmental delay is often defined as a deviation from normative milestones; this may be in terms of delayed cognitive, language, motor and/or socio-emotional development.^18^ The term developmental disabilities covers a range of childhood conditions and is used differently across different settings and cultures.^19^ In this article we define developmental disability as a heterogeneous group of conditions that can impact on the development of children's function (e.g. sensory, cognitive, physical), with a very wide range of effects.^20^ Developmental disability is the most common cause of childhood disability, with an estimated 53 million children <5 y of age living with developmental disabilities globally.^21^ This estimate is based on only six conditions (epilepsy, intellectual disability, vision loss, hearing loss, autism and attention deficit hyperactivity disorder) and on present reporting of these conditions. It is likely therefore that the true number of children with developmental disability is much higher than this estimate, particularly if a broader age range is considered.

The majority of children with developmental disabilities live in LMICs,^21^ and the prevalence is higher among families with high levels of poverty and low education.^27^ However, there remain data gaps for the prevalence, epidemiology and causes of developmental disabilities in LMICs.^28^ One reason for the uncertainty in the estimates is that identification of children with or at risk of developmental delay requires assessment using valid developmental evaluation tools to measure ECD^29^ (Box 1), and these facilities are often not available in LMICs.

**Box 1.** Identification of children with developmental disabilities

In order to meaningfully measure thriving and well-being of children globally, developmental assessment tools need to be culturally relevant and age appropriate and cover the spectrum of developmental domains, including sensory impairments and educational outcomes. Examples of tools with high validity and reliability to measure several developmental domains include the Bayley Scales of Infant and Toddler Development (BSID II or Bayley-III).^22^ Regionally developed instruments include the Malawi Development Assessment Tool^23^ and the Kilifi Developmental Inventory.^24^ However, a recent review found variability in translation, adaptation, piloting and standardisation of tools, with important domains such as vision, hearing, functioning and disability often omitted, which limits holistic understanding of a child's progress.^25^ In addition, no tool covers all domains of development and is accurate and feasible in all contexts.^26^

The impacts of developmental disabilities extend far beyond functional abilities. Children with developmental disabilities and their families are at high risk of social exclusion, exclusion from education and even stigma and violence.^30^ Furthermore, looking after a child with developmental disabilities potentially places an enormous strain on families, and caregivers experience high levels of stress, anxiety, depression, physical exhaustion, stigma and discrimination.^31^ This further increases the risk of mental ill health and social isolation in caregivers. A recent systematic review found caregivers of children with intellectual and developmental disabilities, when compared with caregivers of children without intellectual and developmental disabilities, experienced elevated levels of depressive symptoms (31% vs 7%, respectively) and anxiety symptoms (31% vs 14%, respectively).^32^ There are also substantial costs to childhood disability, both the cost of additional services and resources required by the child and the lost income from parents who are caring for their child. Consequently, childhood disability may exacerbate poverty.^33,34^ However, there is generally a lack of available services and support for children with disabilities and their families, especially in LMICs, which further compound these risks.

## Early intervention for children with developmental disabilities

Evidence is limited, but growing, on the effectiveness of ECD interventions for children at risk of and with developmental delays, particularly in LMICs.^35^ Indeed, many programmes and studies actively exclude children with developmental disabilities, as additional considerations may be required, and children with developmental disabilities may be unable to show progress when using developmental progress as the primary outcome^9,36–38^ (Box 2).

**Box 2.** Inclusion of children with developmental disabilities in clinical trials

Our review of the first 100 titles of registered clinical trials of ECD interventions (Appendix 1), and inclusion of children with disabilities, demonstrated that 50% of the trials exclude children with disabilities, 22% of trials target children with disabilities, 3% of trials target children in general and include children with disabilities and 25% of trials do not specify whether children with disabilities are included or excluded.

Consequently, risks to delayed development are compounded for children with developmental disabilities, as they potentially receive less stimulation and fewer learning opportunities through other health service or care routes.^39^ Exclusion of children with developmental disabilities from ECD thus perpetuates an already fragile cycle of development. We know that early childhood developmental intervention for these children is imperative, but we cannot inform planning and delivery of inclusive services for all children without better research in this area. For example, there are gaps in evidence-based approaches to monitoring and evaluation of ECD projects in LMICs, such as challenges in measurement of outcomes in routine programmes, which limit comparative understanding of impact, and in defining and monitoring quality and coverage.^25^

Early identification of children with developmental disabilities, as well as early childhood intervention (ECI), improves children's opportunities to maximise their developmental potential and functioning as well as their quality of life and social participation.^40,41^ Early identification and intervention are two distinct complementary strands; timely identification of children with developmental disabilities is required for early intervention, which strengthens the cumulative process of development, helping children acquire new skills and behaviours to reinforce and strengthen learning. In addition, some ECIs may have wider benefits for caregivers, such as through establishing support, thus helping build their knowledge, confidence and coping strategies,^32^ with positive impacts for their mental health. However, data are lacking from LMICs and there is a paucity of implementation evidence to guide policymakers and donors.^33^

ECI for children with disabilities can comprise a range of coordinated multidisciplinary services and can take many forms, including hospital- or clinic-based care, school-based programmes, parenting and community support and home-based childhood therapies. In high-resource settings, we know that family-centred interventions are more likely to result in the greatest satisfaction with services and improve psychosocial well-being for the child and caregiver.^42^ With regards to impact, a systematic review of ECIs for children at risk of cerebral palsy demonstrated improved cognitive outcomes up to preschool age and improved motor outcomes during infancy, although variability in interventions limited the identification of which interventions are most effective.^43^ Nevertheless, without such ECIs in LMICs, years lived with disability will be more than 3.3 million.^1^

There are broadly two approaches to providing ECI for children with developmental disabilities, including children with disabilities in mainstream ECD interventions and targeted intervention programmes for children with disabilities. These approaches take many different forms, as they are used to support children and families with different needs. For example, universal programmes in the UK, such as the five mandated health visits for young children, are offered to all families. In contrast, targeted programmes, such as the Disabled Children's Outreach Service (DCOS), are aimed specifically at vulnerable families of children with a disability where the children are at higher risk of poor outcomes in later life.^44^

While both inclusive and targeted efforts for children with disabilities at the level of early childhood centres have increased,^45^ weak country health systems and conflict settings are major impediments to delivering high-quality services.^46^ There remains a need for inclusive approaches for children with developmental disabilities in mainstream services, as well as within specialist ECIs. This means that the role of families can be particularly crucial to fill existing gaps in service availability.

## Case studies of ECI for children with developmental disabilities

A number of case studies have been identified for ECI for children with developmental disabilities. The following have been selected for description, as they illustrate different approaches for children with different developmental disabilities in several LMIC settings.

The WHO has developed Caregiver Skills Training (CST) for caregivers of children with intellectual disabilities.^47,48^ The CST consists of nine group sessions and three home visits. The programme teaches strategies to promote communication and learning and address challenging behaviours. However, sustainable and scalable quality delivery of the group format by a lay facilitator remains a challenge due to limited integration in health systems.^49^ Evidence of effectiveness is currently lacking, but randomised controlled trials are under way in Pakistan (Family Networks [FaNs] for Children with Developmental Disorders and Delays^50^) and Italy, with future trials planned in China, Ethiopia and Kenya.^51^

Interventions that aim to provide contextualised psychological support to caregivers of children with intellectual disabilities include ‘Titukulane’, a community group intervention that aims to reduce mental health problems among the parents of affected children.^52^ This community-based intervention consists of eight modules that have been developed and piloted to help parents cope with the challenging role of caring for a child with intellectual disabilities.

Learning through Everyday Activities with Parents (LEAP-CP) is a family-centred intervention delivered peer to peer at home during 30 weekly 2h visits that aims to improve the mobility of children with cerebral palsy.^53^ Visits include therapeutic modules (goal-directed active motor and cognitive strategies and LEAP-CP games) and parent education. Randomised controlled trials are currently under way in India.^54^ The trial also provides nutrition and health support to all families in the study, which may influence the findings.

The London School of Hygiene & Tropical Medicine (UK) has developed three caregiver group interventions under the ‘Ubuntu’ umbrella (resources available from www.ubuntu-hub.org). The interventions consist of 10 sessions, the content of which includes information about essential care practices, such as feeding, positioning, communication and play, offered through a local support group format. ‘Getting to know cerebral palsy’ was developed as a resource to empower families using a participatory approach at the community level.^31,55^ The ABAaNA Early Intervention Programme (EIP) was developed in response to a recognised need to support families of very young children (<2 y) with an evolving developmental disability.^56^ ‘Juntos’ was developed for children with congenital Zika syndrome and their families in Latin America and integrates a strengthened component on caregiver emotional well-being, arguably fundamental to a child's early development.^57–60^

Interventions for children with autism spectrum disorder include PASS, a parent-mediated intervention for autism spectrum disorder in India and Pakistan.^61^ The intervention uses video feedback methods to address parent–child interaction and was adapted for delivery by non-specialist workers. As PASS is focused on improving a child's social communication, common mental health comorbidities such as sleep difficulties will be important to integrate into wider intervention programmes.

These examples provide good case studies of diverse interventions for different children with developmental disabilities in different low-resource settings. These case studies indicate that in LMICs, the gap in meeting the holistic needs of children with developmental disabilities may be addressed through the use of community-based group interventions facilitated by trained and supervised health or peer support workers. Commonality is the focus on caregiver involvement, which is critical, particularly where there are few health services. Yet formal evaluation of their effectiveness and cost-effectiveness is lacking, in addition to limited implementation with education and social welfare, which hampers scaling of these services.

## The case for action

The number of children with developmental disabilities is large and the impacts on the child and family are extensive. There are valuable lessons learned from case studies, yet there remains insufficient progress in ECI for children with developmental disabilities and unmet needs are widespread. The causes of this gap are complex and diverse. An important reason is that in many settings health services are often fragile, poorly coordinated and overstrained, with concerns about the availability and quality of healthcare workers capable of delivering the intervention. Health systems gaps are particularly important in fragile states, including those affected by war and famine, as they experience many competing pressing needs. Furthermore, the policy agenda supporting a focus on children with developmental disabilities is weak internationally and nationally in many cases, limiting the priority given to this issue and the availability of funding for developing services. Ensuring inclusive education is a clear responsibility for United Nations member states under international treaties and Sustainable Development Goal 4, to ‘ensure inclusive, equitable quality education for all’. However, investing in inclusion prior to schooling is not mandated and consequently becomes optional. Cultural challenges also exist, such as widespread stigma and discrimination around children with disabilities and their families.^62^ Finally, the evidence base on needs for and effectiveness of services is currently weak and needs to be strengthened. Enhancing environments that provide equal opportunities for children with developmental disabilities for ECI therefore requires a systems approach with global collaboration.

Accordingly, priorities for future research to ensure that all young children reach their development potential include assessment of the effect of interventions for children with developmental disability and their families in different low-resource settings. Further identification of barriers to accessing general services (e.g. primary healthcare) as well as specialist services is also required, as poverty remains a major issue for affected families in LMICs. Furthermore, studies that identify how to maximise the reach and cost-effectiveness of ECD interventions for children with developmental disabilities are warranted. Evaluation of how these interventions can be embedded within health systems are needed to strengthen the service delivery strategies. Global collaboration in these efforts are required in research, and critical steps include providing best evidence on practices to improve knowledge and skills at local levels to avoid children with developmental disabilities being turned away from existing services and evidence of ‘what works’ to provide sustainable, inclusive ECD interventions with impact in resource-constrained settings. We call for international research communities, including funders, to adopt a systematic approach for better evidence.

## Conclusion

ECD interventions are aimed at improving the development of children. However, children with developmental disabilities are often excluded from these programmes, even though they have the greatest need for support. There is still a dearth of research about what interventions are effective in improving outcomes for this marginalised group and an even greater lack of evidence on cost-effectiveness and what can be successfully implemented at scale. A two-pronged approach is likely to be optimal, encouraging the inclusion of children with disabilities in mainstream ECD programmes, while also offering targeted approaches, most likely through caregivers. We call for global collaboration among international research communities, including funders, to adopt a systematic approach to strengthening the available evidence base of interventions for children with developmental disabilities and their families. We call for greater attention for this marginalised group, to prioritise public policies and hold governments accountable to ensure that multisectoral services centred around the child and his/her family are provided during this crucial time. This will contribute to ensuring that all children have an early foundation for optimal development, a key factor in equitable long-term health.

## Appendix

**Table A1.** Completed clinical trials with a focus on developmental outcomes

	Title	Start date	Country	Target children with disabilities	Includes children with disabilities	Does not include or exclude	Excludes children with disabilities
1	The Pakistan Early Childhood Development Scale Up Trial	2009	Pakistan				1
2	Iron Treatment for Young Children With Non-anemic Iron Deficiency	2012	Canada				1
3	Project Grow Smart: Intervention Trial of Multiple Micronutrients and Early Learning Among Infants in India	2012	India				1
4	Early Child Development and Nutrition in Guatemala	2015	Guatemala				1
5	Strong Families, Thriving Children “Sugira Muryango”_Activity C	2018	Rwanda			1	
6	Implementation and Adoption of Care for Child Development in Day Care Centers	2015	Lebanon				1
7	CASITA Intervention for Children at Risk of Delay in Carabayllo, Peru	2013	Peru			1	
8	Family Inclusive Early Brain Stimulation	2014	Nigeria				1
9	Applying Mindfulness for Economically Disadvantaged Families	2016	Hong Kong				1
10	Promoting Child Development Practices in the First Year of Life Through a Video Administered at Two Different Times	2008	Italy			1	
11	Promoting Early School Readiness in Primary Health Care	2005	USA				1
12	Improving Early Childhood Development in Zambia	2014	Zambia			1	
13	Early Intervention for Developmental Delay	2014	Taiwan				1
14	A Family Centered Intervention to Promote Optimal Child Development	2013	USA			1	
15	Family Strengthening Intervention for Early Childhood Development (ECD)	2014	Rwanda			1	
16	Screening for Therapy and Empowering Parents: A Pilot Study	2015	USA				1
17	Alliance for Family Strengthening: Improved Early Childhood Development in Rwanda	2017	Rwanda			1	
18	Efficacy of Tools of the Mind for Enhancing Self-Control in Preschoolers	2012	Canada				1
19	Early Literacy Promotion Intervention	2016	USA				1
20	Zinc and Biobehavioral Development in Early Childhood	2004	Peru				1
21	The Effect of a Cash Transfer Program and Preventive Nutrition Packages on Household Welfare and Child Nutritional Status in Mali	2014	Mali				1
22	Enhancing Ugandan HIV-Affected Child Development With Caregiver Training	2012	Uganda				1
23	Early Intervention for Preterm Infants	2006	Taiwan				1
24	Effect of Improving Caregiving on Early Mental Health	2000	Russia			1	
25	Testing the Effectiveness of Telephone-based Early Childhood Developmental Screening	2015	USA		1		
26	Early Family-Centered Prevention of Drug Use Risk (Aka Early Steps)	2003	USA			1	
27	Effect of Power Wheelchairs on the Development and Function of Young Children With Severe Physical Disabilities	2002	USA	1			
28	Effect of Community Based Depression Management and Child Development	2014	Bangladesh				1
29	Play and Pre-Literacy Among Young Children	2015	Canada				1
30	Social and Communication Outcomes for Young Children With Autism	2009	USA		1		
31	The Anemia Control Program: Early Intervention	1992	Chile				1
32	Early Psychosocial Stimulation Program for Children of Depressed Mothers	2009	Pakistan			1	
33	Promoting Infant Mental Health in Foster Care	2007	USA			1	
34	Addressing Systemic Health Disparities in Early Identification and Treatment of Autism Spectrum Disorder (ASD): ABCD Project	2014	USA		1		
35	The MOM Program at the Children's Hospital of Philadelphia	2001	USA			1	
36	Translating Evidence Based Developmental Screening Into Pediatric Primary Care	2008	USA				1
37	Improving Parental Psychosocial Functioning and Early Developmental Outcomes in Children With Sickle Cell Disease	2014	West Indies			1	
38	Promoting Healthy Development With the Recipe 4 Success Intervention	2013	USA			1	
39	Long Term Effect of Early Iron Supplementation and Psychosocial Stimulation on Growth and Development of Iron-deficient Anaemic Infants	2015	Bangladesh			1	
40	Reduce Childhood Maltreatment and Promote Development	2015	Bangladesh				1
41	The Impact of Cash and Food Transfers Linked to Preschool Enrollment on Child Nutrition and Cognitive Outcomes	2010	Uganda			1	
42	Effects of Family-Centered Intervention for Preterm Infants at Preschool Age	2015	Taiwan				1
43	Effectiveness of Parent-Child Interaction and Emotion Development Therapy in Treating Preschool Children With Depression	2007	USA				1
44	The Effects of Iodized Salt on Cognitive Development in Ethiopia	2011	Ethiopia		1		
45	An Intervention for Enhancing Early Attachment in Primary Health Care	2013	Chile				1
46	The MOM Program: 5 Year Follow-up Study of a Home Visiting Program at the Children's Hospital of Philadelphia	2004	USA				1
47	Small Step Intervention for Infants With Cerebral Palsy and Other Neurodevelopmental Disorders	2014	Sweden	1			
48	Intervention Effects of Intensity and Delivery Style for Toddlers With Autism	2008	USA		1		
49	Motivational Interviewing to Increase Parent Engagement in Preventive Parenting Programming	2013	USA				1
50	Intensive Intervention for Toddlers With Autism (EARLY STEPS)	2013	USA		1		
51	Optimizing Social and Communication Outcomes for Toddlers With Autism	2008	USA		1		
52	Primary Prevention of Allergic Disease in Early Child by *Lactobacillus reuteri*	2001	Sweden			1	
53	Intervention for Toddlers at Risk for Autism Spectrum Disorders (ASD)	2008	USA		1		
54	Promoting Development in Toddlers With Communication Delays	2007	USA				1
55	Early Intervention, Supervision, Quality and Outcome in ASD	2013	Sweden			1	
56	Differential DNA Methylation as a Function of a Parenting Intervention	2013	USA				1
57	Early Connections, Early Detection and Intervention in Infants at Risk for Autism	2008	USA		1		
58	Early Characteristics of Autism	2003	USA		1		
59	School- and Home-Based Early Intervention for Toddlers With Autism	2003	USA		1		
60	Follow-up of Families in Early Preventive Intervention	2000	USA			1	
61	Parent Training Program for Preschool Children With Autism Spectrum Disorders	2015	Taiwan		1		
62	RESPECT-PLUS: Services for Infants With Prenatal Opiate Exposure	2013	USA				1
63	Early Nutritional Intervention in Patients With Autism Spectrum Disorders	2010	Qatar		1		
64	Maximizing Language Development in Children With Hearing Loss	2013	USA				1
65	Mother and Child Education Program in Palestinian Refugee Camps	2014	Lebanon			1	
66	Early Intervention and Autism: Transformation From Research to Practice Through a Competency Based Model	2017	Sweden		1		
67	Mindfulness Training and Parent-coaching Interventions for Autism Spectrum Disorder	2015	USA		1		
68	Impact of an Intervention Program on Parenting Stress After Preterm Birth	2006	France				1
69	Efficacy Trial of the Kids in Transition to School (KITS) Program for Children With Developmental Disabilities and Behavioral Problems	2008	USA				1
70	Social-emotional Under 4’s Screening & Intervention S.U.S.I.	2016	USA		1		
71	H3: Healthy Minds, Healthy Children, Healthy Chicago Project Evaluation	2014	UK			1	
72	Reproducibility Inter-session of the Measurement Elastography of the Passive Stiffness of Medial Beams of Gastrocnemius Muscle of the Hemiplegic Cerebral Child	2017	France	1			
73	Transition to Scale of Nutrition and Psychosocial Stimulation Program for Malnourished Children	2014	Bangladesh				1
74	Electronic Patient-reported Outcomes (e-PROs) in Early Intervention	2016	USA	1			
75	Iron Deficiency Anemia and Psychosocial Stimulation	2007	Bangladesh				1
76	Omega Tots: A Randomized, Controlled Trial of Long-chain Polyunsaturated Fatty Acid Supplementation of Toddler Diets and Developmental Outcomes	2012	USA			1	
77	Zinc, Iron, Vitamin A and Psychosocial Care for Child Growth and Development	1998	Indonesia				1
78	Middle Ear Disease Before Age 3, Treatment With Ear Tubes, and Literacy and Attentional Abilities at Ages 9 to 11	2002	USA				1
79	The Effect of a Deworming Intervention to Improve Early Childhood Growth and Development in Resource-poor Areas	2014	USA				1
80	Comparing Parent-Implemented Interventions for Toddlers With Autism Spectrum Disorders	2007	USA		1		
81	Strengthening Families and Reducing Risk Thru Developmental and Legal Collaboration	2011	USA				1
82	Social Cognitive Development in Young Children With Autism	2012	USA		1		
83	Evaluation of the Healthy Families Alaska Program	1999	USA			1	
84	Initial Efficacy Study of Supporting Play, Exploration, & Early Development Intervention	2011	USA				1
85	Healthy Habits, Happy Homes: An Intervention to Improve Household Routines for Obesity Prevention	2011	USA				1
86	Age 12 Follow-up of Early Preventive Intervention (Memphis)	2003	USA			1	
87	Project ASPIRE Efficacy Pilot: Achieving Superior Parental Involvement for Rehabilitative Excellence	2009	USA				1
88	Interventions for Communication in Autism Network	2012	USA		1		
89	The Effects of a Parental Intervention on Electronic Media Exposure and Sleep Patterns in Adolescents	2011	Israel				1
90	A Trial of Sertraline in Young Children With Autism Spectrum Disorder	2015	USA		1		
91	A Randomized Controlled Trial of PCIT-ED for Preschool Depression	2014	USA				1
92	Psychomotor Therapy for Very Premature Infants	2007	France				1
93	A Website to Teach Children Safety With Dogs	2015	USA				1
94	Early Physical Therapy Intervention in Preterm Infants	2017	Spain				1
95	The Children in Action Feasibility Study	2007	USA				1
96	Development of Appetite Measuring Tool and Appetite Status of Stunted Children	2016	Bangladesh				1
97	Early Pharmacotherapy Aimed at Neuroplasticity in Autism: Safety and Efficacy	2004	USA		1		
98	Study and Development of Application Models of “Therapeutic Education to the Patient” (TEP) in Asthmatic Children	2007	Italy			1	
99	Development and Effectiveness of Home-based Programs for Preschool Children With Developmental Delay	2017	USA				1
100	Digital Literacy Promotion	2016	Bangladesh				1
	TOTAL			4	21	25	50
